# Galanin is a potent modulator of cytokine and chemokine expression in human macrophages

**DOI:** 10.1038/s41598-019-43704-7

**Published:** 2019-05-10

**Authors:** Andreas Koller, Susanne Maria Brunner, Rodolfo Bianchini, Andrea Ramspacher, Michael Emberger, Felix Sternberg, Sandra Schlager, Barbara Kofler

**Affiliations:** 1 Research Program for Receptor Biochemistry and Tumor Metabolism, Department of Pediatrics, University Hospital of the Paracelsus Medical University, Salzburg, Austria; 2 Research Program for Ophthalmology and Glaucoma Research, Department of Ophthalmology and Optometry, University Hospital of the Paracelsus Medical University, Salzburg, Austria; 3 Laboratory for Pathology Weger, Emberger, Salzburg, Austria; 4 Department of Blood Group Serology and Transfusion Medicine, University Hospital of the Paracelsus Medical University, Salzburg, Austria; 5 Department of Comparative Medicine, The Interuniversity Messerli Research Institute, University of Veterinary Medicine Vienna, Medical University of Vienna, University Vienna, Vienna, Austria; 6 Department of Biomedical Sciences, Unit of Physiology and Biophysics, University of Veterinary Medicine, Vienna, Austria

**Keywords:** Gene regulation in immune cells, Neuroimmunology

## Abstract

The regulatory peptide galanin is broadly distributed in the central- and peripheral nervous systems as well as in non-neuronal tissues, where it exerts its diverse physiological functions via three G-protein-coupled receptors (GAL_1-3_-R). Regulatory peptides are important mediators of the cross-communication between the nervous- and immune systems and have emerged as a focus of new therapeutics for a variety of inflammatory diseases. Studies on inflammatory animal models and immune cells revealed both pro- and anti-inflammatory functions of galanin. Here, we probed specific immune-related functions of the galanin system and found galanin and GAL_1_-R and GAL_2_-R mRNA to be expressed in a range of human immune cells. In particular, macrophages displayed differentiation- and polarization-dependent expression of galanin and its receptors. Exposure to exogenous galanin affected the cytokine/chemokine expression profile of macrophages differently, depending on their differentiation and polarization, and mainly modulated the expression of chemokines (CCL2, CCL3, CCL5 and CXCL8) and anti-inflammatory cytokines (TGF-β, IL-10 and IL-1Ra), especially in type-1 macrophages. Cytokine/chemokine expression levels in interferon-gamma- and lipopolysaccharide-polarized macrophages were upregulated whereas in unpolarized macrophages they were downregulated upon galanin treatment for 20 hours. This study illuminates the regulation of important cytokines/chemokines in macrophages by galanin, depending on specific cell activation.

## Introduction

Bidirectional communication between the neuroendocrine- and immune systems is of paramount importance for maintaining physiological equilibrium between pro- and anti-inflammatory conditions during inflammation and homeostasis^1,2^. Diverse regulatory peptides, which could act as hormones, neurotransmitters and neuromodulators such as vasoactive intestinal peptide (VIP), substance P (SP), corticotropin-releasing hormone (CRH) and neuropeptide Y (NPY), are released not only by nerve terminals but also by diverse immune cells to modulate various immune responses directly or indirectly^1,2^.

Another member of these regulatory peptides is galanin, which was first described in the early 1980s^3^. The 30/29 amino acid (human/rodent) peptide is widely distributed in the central- and peripheral nervous systems and in non-neuronal tissues as well^4^. Galanin is processed from a 123/124 amino acid (human/rodent) precursor^5^ and plays a role in diverse physiological functions like feeding, nociception, learning, memory, stress and inflammation^4^. Galanin exerts its functions via three G-protein coupled receptors (GAL_1-3_-R), exhibiting highest affinity for GAL_1_-R and GAL_2_-R^4^. In 1999, another endogenous galanin receptor ligand was described, which was named galanin-like peptide (GALP) due to its sequence homology to galanin^6^. GALP is linked with male sex behavior in rats and mice^7,8^, body weight/food intake in mice^9^, and thermoregulation in rats^10^. GALP is expressed mainly in the central nervous system and the peptide is able to bind to all three galanin receptors, with the highest affinity to GAL_3_-R^4^. Recently, a newly discovered endogenous 14 amino acid regulatory peptide, named spexin, was reported to be selective for GAL_2_-R and GAL_3_-R, and to have higher affinity to GAL_3_-R than galanin^11,12^. Spexin is highly expressed in the nervous system and non-neuronal tissue, especially in epithelial cells^13^. Low serum levels of spexin were reported to negatively correlate with obesity^14,15^ and to suppress food intake^16^.

Galanin receptor signaling leads to a reduction of cAMP concentration and inactivation of protein kinase A (PKA) via the G_i/o_ signaling cascade or activation of protein kinase C (PKC) via G_q/11_ signal transduction^4^. Both enzymes, PKA and PKC, are important regulators of immune cell functions^17–22^. An indispensable role of the galanin system in immune regulation has been underpinned by several *in vitro* and *in vivo* studies. For example, high amounts of galanin were found in porcine lymphoid organs^23^. Furthermore, galanin is produced in peripheral tissues such as the skin during inflammation^24^. In addition, GAL_1_-R was reported to be upregulated in the acute inflammatory phase in peripheral tissues^25–27^. The immune regulatory capacity of released galanin and its receptors was proven in various animal models of inflammatory diseases, including inflammatory bowel disease (IBD), arthritis, dermatitis, psoriasis and pancreatitis^28–34^. Recently, our laboratory demonstrated a pro-inflammatory role of GAL_3_-R, but not GAL_2_-R, in an imiquimod-induced psoriasis mouse model. GAL_3_-R KO mice showed reduced levels of erythema, scaling, and thickening of the skin during the progression of inflammation. Furthermore, GAL_3_-R KO mice exhibited reductions in spleen weight, vascularization, myeloperoxidase (MPO) activity, neutrophil infiltration, and expression of crucial cytokines involved in psoriasis, including IL-17A, IL-22, IL-23 and TNFα^35^. At the cellular level, we demonstrated that human natural killer (NK) cells express GAL_2_-R and that galanin modulates interferon-gamma (IFNγ) production and release by NK cells upon IL-12 and IL-18 stimulation^36^. In addition, we reported galanin-directed modulation of polymorphonuclear neutrophil (PMN) sensitivity toward the chemokine CXCL8 (IL-8)^37^. Furthermore, we observed significant induction of galanin expression and concomitant reduction of GAL_2_-R expression upon granulocyte-macrophage colony-stimulating factor (GM-CSF)-induced differentiation of human monocytes to macrophages^36^. Immune cells can be roughly divided by their cell lineage into myeloid and lymphoid cells. The myeloid cell lineage includes mainly neutrophils, eosinophils, basophils, monocytes, macrophages, dendritic cells and mast cells, whereas the lymphoid cell lineage includes B cells, T cells and innate lymphoid cells (ILC), including NK cells. Of high interest are macrophages, because their level of galanin expression is more than 100-fold higher compared to any other type of galanin-positive immune cell, such as monocytes^36^. Thus, galanin could affect macrophages via an autocrine mechanism, but also other cell types and tissues via paracrine and endocrine signaling.

As macrophages are key players in body homeostasis, innate immunity and initiation of adaptive immune responses, the diverse subtypes of macrophages are classified according to their different phenotypes and functional abilities^38^. Macrophages are heterogeneous and pleiotropic cells found in virtually all tissues of adult mammals. Their specialization in particular microenvironments explains their heterogeneity. For tissue-resident macrophages, the subdivision is firstly based on their origin: embryonic or monocyte-derived. Macrophages are further subdivided according to their tissue location, such as osteoclasts (bone), alveolar macrophages (lung), microglia (brain), Kupffer cells (liver) and Langerhans cells (skin). Although these various macrophages are exposed to different microenvironments, they share certain critical functions like defense against pathogens, clearance of cellular debris, immune surveillance and wound healing^38^. During the inflammatory process, macrophages can differentiate roughly into either M1 or M2 cells. M1 macrophages are associated with T_h_1 responses, killing intracellular pathogens and tumor resistance. M1 macrophages increase the production of reactive oxygen species (ROS), nitric oxide (NO), MPO and inflammatory cytokines. M2 macrophages are subdivided into types M2a (T_h_2 responses, allergy, killing and encapsulation of parasites), M2b (T_h_2 activation and immunoregulation), M2c (immunoregulation, matrix deposition and tissue remodeling) and M2d (wound healing, angiogenesis)^39–42^.

As galanin expressly plays a role in various inflammatory processes and diseases and as other regulatory peptides were reported to modulate immune responses^1^, the direct effects of galanin and its receptors on various immune cells have to be demonstrated. The aim of the present study was first to examine the regulation of the galanin system during macrophage differentiation and polarization. Secondly, we aimed to elucidate the impact of galanin on macrophage cytokine/chemokine expression and secretion profiles.

## Results

### Expression and regulation of the galanin system in immune cells

Recently, we reported that peripheral monocytes express moderate levels of galanin, which increased upon GM-CSF-induced differentiation of monocytes to macrophages (M0-GM-Mφ)^36^, whereas we detected no galanin expression in PMNs and NK cells^36,37^. In the present study, we extended this analysis to human B cells and CD4^+^ and CD8^+^ T cells, and all were found to express galanin at levels similar to monocytes (Table 1). Interestingly, all immune cells showed low mRNA levels of spexin, whereas no GALP expression was observed (Table 1).

**Table 1:** Relative expression levels of the galanin system in human immune cells.

	Galanin	GALP	Spexin	GAL_1_-R	GAL_2_-R	GAL_3_-R
PMNs	−^a^	−	+	−^a^	++^a^	−^a^
NK cells	−^b^	−	+	−^b^	+^b^	−^b^
B cells	+	−	+	−	++	−
T cells CD4^+^	+	−	+	−	+	−
T cells CD8^+^	+	−	+	−	+	−
Monocytes	+^b^	−	+	− ^b^	++^b^	−^b^
M0-GM-Mφ	+++/+++^b^	−	+	+/−^b^	+/+^b^	~/−^b^
M0-M-Mφ	+++	−	+	+	+	~

Expression levels were normalized to the housekeeping gene RPL27 (ΔΔCq). +++ = ΔΔCq > 0.03125. ++ = ΔΔCq. > 0.000977 – ≤ 0.03125. + = ΔΔCq > 0.0000305. – ≤ 0.000977.

~ = ΔΔCq > 0 − ≤ 0.0000305.

− = no expression detected.

n = 3–10 (GALP: T cells CD4^+^, n = 2; T cells CD8^+^, n = 1).

^a^Reported in Locker, *et al*.^37^.

^b^Reported in Koller, *et al*.^36^.

**Figure 1:**
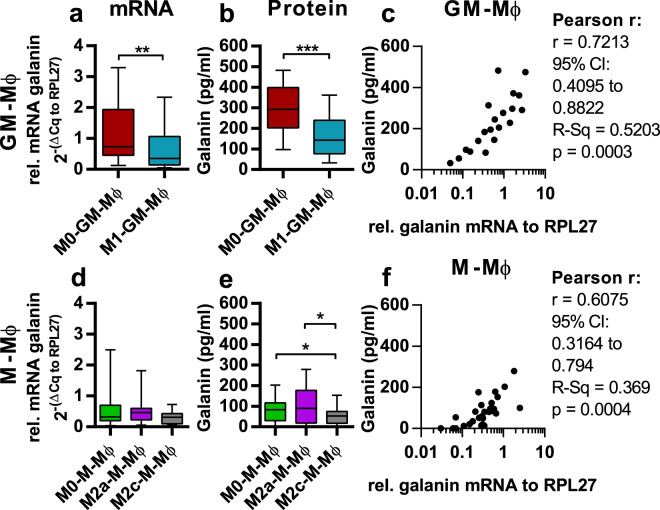
Galanin peptide concentrations in the supernatant of macrophages and corresponding mRNA levels. Galanin mRNA levels were analyzed in GM-Mφ (**a**) and M-Mφ (**d**) by qPCR. Supernatants of GM-Mφ and M-Mφ were analyzed by ELISA for full-length galanin with or without polarization with IFNγ + LPS (M0-GM-Mφ and M1-GM-Mφ) (**b**), IL-4 or IL-10 (M0-M-Mφ, M2a-M-Mφ and M2c-M-Mφ) (**e**) *in vitro* [n = 10]. The values are represented in box and whiskers plot format – min to max; n = 10. *p < 0.05, **p < 0.01, ***p < 0.001. Correlation of galanin mRNA expression and peptide secretion in GM-Mφ [n = 20] (**c**) and M-Mφ [n = 30] (**f**) were computed with Pearson’s correlation coefficient.

As macrophages expressed high amounts of galanin mRNA, we analyzed the amount of secreted galanin by macrophages (Fig. 1). All macrophage subtypes secreted full-length galanin and the amount of secreted galanin correlated with the mRNA expression level (Fig. 1c,f). To determine how polarization affects galanin expression and secretion, we polarized macrophages to M1-GM-Mφ (with IFNγ + LPS), M2a-M-Mφ (with IL-4) and M2c-M-Mφ (with IL-10). To verify the polarization status, the cytokine/chemokine expression profile of the macrophage subtypes was quantified by qPCR (Supplementary Fig. S1). Overall, we found type-1 macrophages (M0-GM-Mφ and M1-GM-Mφ) express and secrete higher amounts of galanin than type-2 macrophages (M0-M-Mφ, M2a-M-Mφ and M2c-M-Mφ) independent of their polarization status (Fig. 1). However, we found that polarization also has an effect on galanin expression and secretion (Fig. 1). M1-GM-Mφ showed significantly reduced galanin mRNA expression and secretion compared to M0-GM-Mφ (Fig. 1a,b). M2c-M-Mφ exhibited a significant reduction of galanin secretion, but its mRNA level was unchanged compared to unpolarized M0-M-Mφ (Fig. 1d,e). M2a-M-Mφ showed no difference in the expression and secretion levels of galanin compared to M0-M-Mφ (Fig. 1d,e).

**Figure 2:**
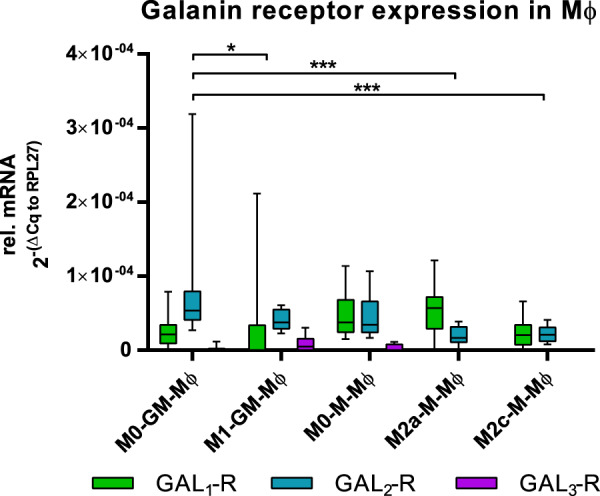
Relative (ΔΔCq to housekeeping gene RPL27) galanin receptor mRNA levels in macrophages with or without polarization for 20 hours. The values are represented in box and whiskers plot format – min to max; n = 10. *p < 0.05, ***p < 0.001.

The galanin system comprises three receptors, GAL_1-3_-R, which exhibit different binding affinities to galanin ligands and are linked to diverse inflammatory processes. Therefore, we screened diverse immune cells for their galanin receptor expression. We previously showed that PMNs, NK cells and monocytes express GAL_2_-R but not GAL_1_-R or GAL_3_-R^36,37^. In the present study, we observed that human B cells and CD4^+^/CD8^+^ T cells also express GAL_2_-R but not GAL_1_-R or GAL_3_-R (Table 1). Furthermore, M0-GM-Mφ and M0-M-Mφ showed consistent GAL_1_-R and GAL_2_-R expression and sporadically low levels of GAL_3_-R (Table 1, Fig. 2). A more detailed analysis of macrophage subtypes revealed that GAL_2_-R expression is significantly lower in polarized macrophages (M1-GM-Mφ, M2a-M-Mφ and M2c-M-Mφ) compared to M0-GM-Mφ (Fig. 2). We observed no significant difference of GAL_1_-R expression in macrophage subtypes (Fig. 2). GAL_3_-R was only sporadically expressed in four of 10 donors at very low levels (Fig. 2).

**Figure 3:**
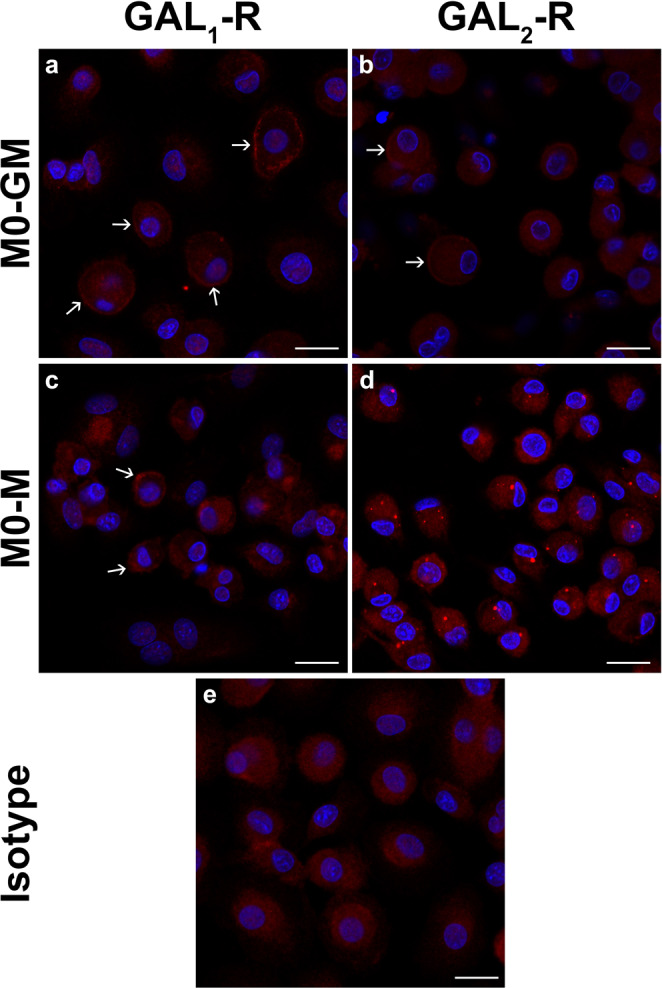
Representative immunofluorescence microscopy of GAL_1_-R and GAL_2_-R in macrophages. M0-GM-Mφ (**a**,**b**) and M0-M-Mφ (**c**,**d**) were analyzed with antibodies against GAL_1_-R (**a**,**c**) and GAL_2_-R (**b**,**d**). IgG isotype control (**e**) was used to identify unspecific binding. The receptor analysis was performed in macrophages of two different donors. Arrows indicate galanin receptor positive macrophages. Scale bar = 20 µm.

After detection of GAL_1_-R and GAL_2_-R mRNA expression in macrophages, we analyzed the protein expression of both receptors in M0-GM-Mφ and M0-M-Mφ by immunofluorescence microscopy using anti-galanin receptor antibodies which were recently validated for specificity in our laboratory^43,44^. Macrophage cell clusters were excluded from analysis as the IgG isotype control showed false positive staining at cell-to-cell contacts (data not shown). Therefore, only single cells were evaluated for their galanin receptor expression. Interestingly, only about 30–40% of M0-GM-Mφ showed a weak but clear positive cell membrane staining of GAL_1_-R (Fig. 3a). Less than 1% of M0-GM-Mφ exhibited a very weak membrane-associated GAL_2_-R staining (Fig. 3b). No definite GAL_1_-R staining was found in M0-M-Mφ (Fig. 3c). M0-GM-Mφ revealed a clear intracellular staining of GAL_2_-R in form of dots mostly located next to the nucleus (Fig. 3d). IgG isotype control did not show specific cell staining (Fig. 3e).

**Figure 4:**
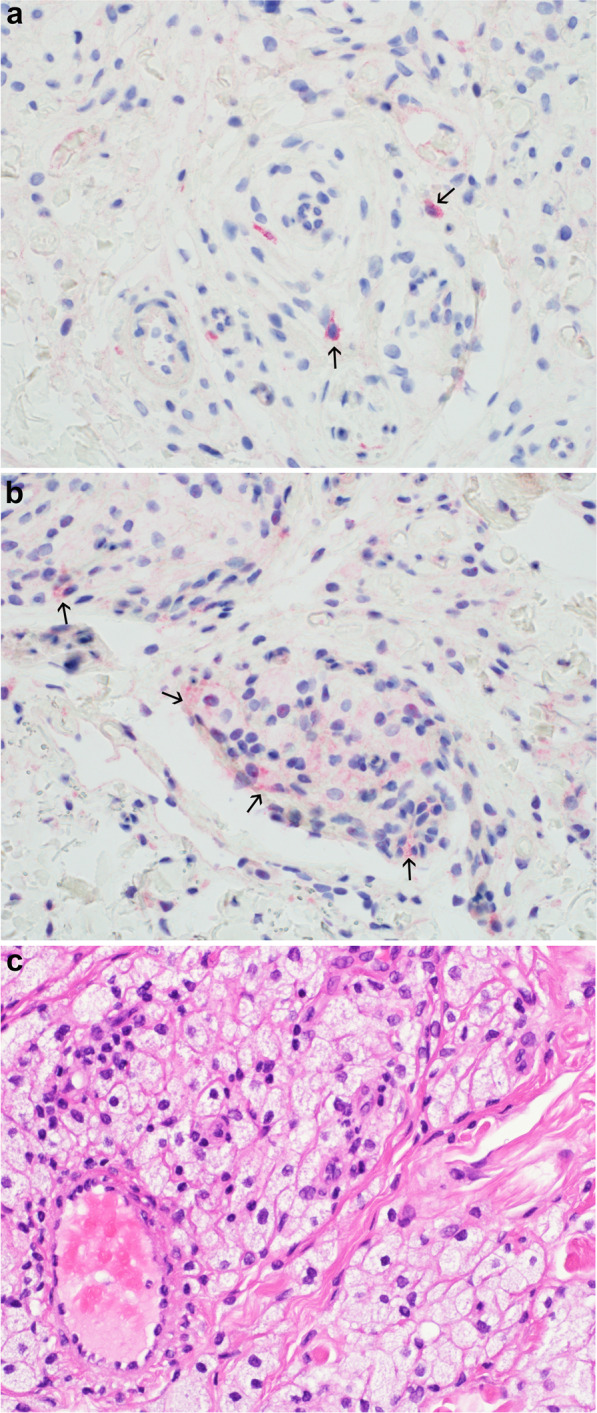
Immunohistochemical staining of human xanthelasma against human GAL_1_-R (**a**) and GAL_2_-R (**b**). Positive staining is observed on xanthelasma macrophages. Hematoxylin and eosin staining of the xanthelasma (**c**). Arrows indicate galanin receptor positive macrophages.

To evaluate the *in vivo* relevance of GAL_1_-R and GAL_2_-R expression on macrophages we determined galanin receptor protein expression in human xanthelasma deposits by immunohistochemistry. A xanthelasma, also referred as xanthoma, is a cluster of foam cells in the connective tissue of the skin. The foam cells are formed from macrophages accumulating lipids by phagocytosis^45^. We found membrane-associated GAL_1_-R (Fig. 4a) as well as GAL_2_-R (Fig. 4b) staining on macrophages in the xanthelasma deposits. In agreement with immunofluorescence microscopy, not all but some macrophages were positive for receptor expression. Interestingly, the more membrane-associated staining of GAL_1_-R in the xantelasma macrophages is similar to the staining observed in the monocyte derived M0-GM-Mφ. Whereas the vesicular staining of GAL_2_-R in the xantelasma macrophages is similar to the staining observed in the monocyte derived M0-M-Mφ.

### Galanin modulates cytokine/chemokine expression in macrophages

**Figure 5:**
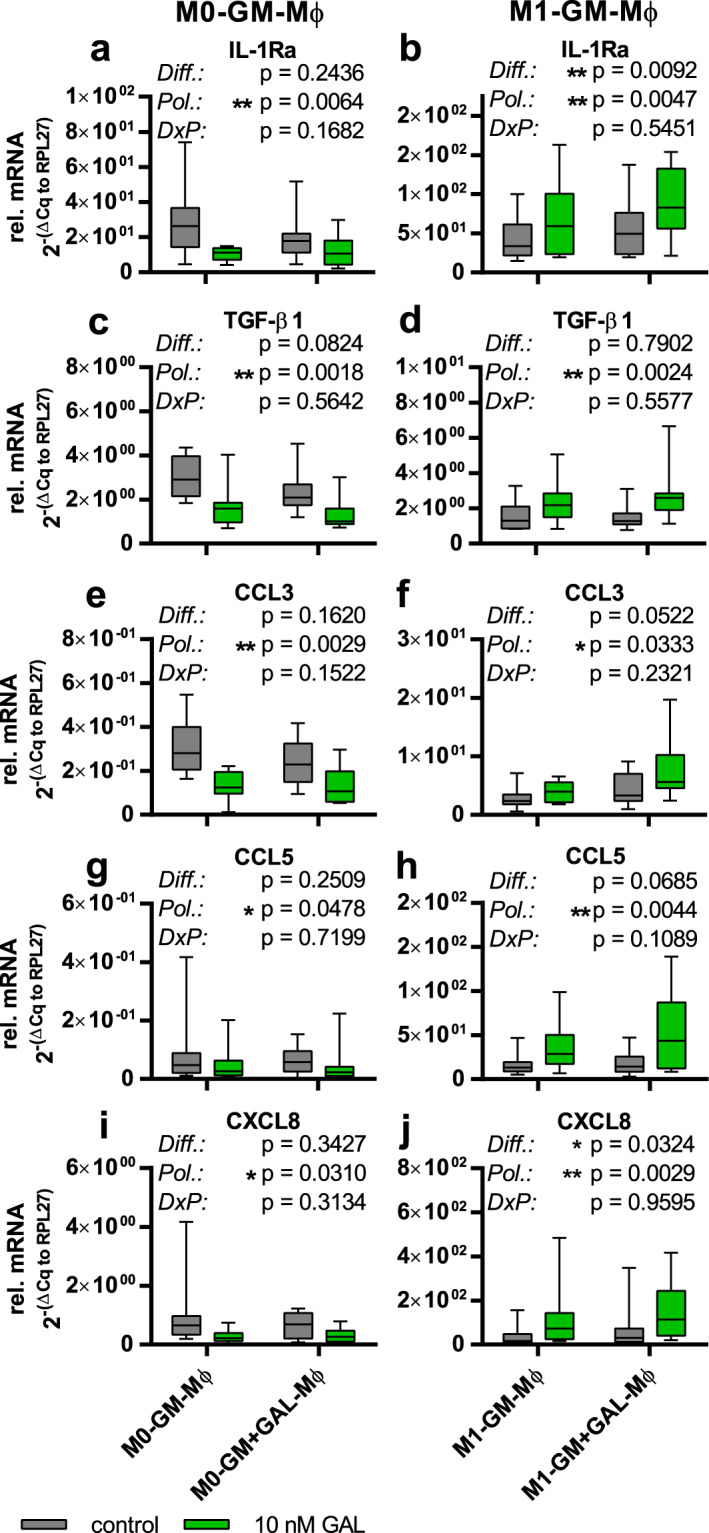
Relative (ΔΔCq to housekeeping gene RPL27) mRNA levels of diverse subtypes of macrophages. Macrophages differentiated with GM-CSF without (M0-GM-Mφ) or with galanin (M0-GM + GAL-Mφ) were further treated with galanin (**a**,**c**,**e**,**g**,**i**) or polarized with IFNγ + LPS (M1-GM-Mφ/M1-GM + GAL-Mφ) without or with galanin (**b**,**d**,**f**,**h**,**j**) for 20 hours and analyzed for expression levels of IL-1Ra (**a**,**b**), TGF-β1 (**c**,**d**), CCL3 (**e**,**f**), CCL5 (**g**,**h**) and CXCL8 (**i**,**j**). The values are represented in box and whiskers plot format – min to max; n = 10. *p < 0.05, **p < 0.01; Diff. = Differentiation; Pol. = Polarization; DxP = interaction of differentiation and polarization.

**Figure 6:**
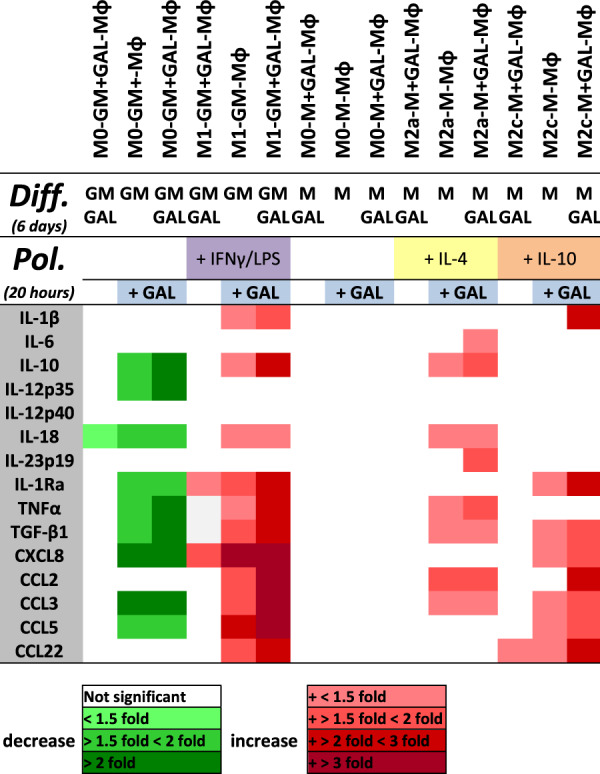
Heat map representing the fold change of cytokine/chemokine expression in macrophage subtypes upon galanin treatment during differentiation (main factor a) and/or polarization (main factor b) to respective control samples differentiated and polarized without galanin (Supplementary Fig. S1). Values are colored when a main factor or an interaction of the main factors (differentiation or polarization) was significant by two-way ANOVA.

As galanin is secreted by macrophages and because monocytes/macrophages express galanin receptors, we investigated the effect of galanin on the cytokine/chemokine profile of macrophages during differentiation and polarization. To examine the impact of galanin on macrophage differentiation and subsequent polarization, we differentiated monocytes into macrophages with GM-CSF or macrophage colony-stimulating factor (M-CSF) in the presence or absence of 10 nM galanin. Afterwards, the differentiation factors were removed and the macrophages were either left untreated, treated with 10 nM galanin (for 20 hours) or polarized with appropriate inducers (IFNγ + LPS or IL-4 or IL-10) plus 10 nM galanin (or no galanin) for 20 hours. The expression data of cytokines/chemokines were analyzed in terms of: 1) differentiation of monocytes in the presence or absence of galanin, and 2) polarization of macrophages in the presence or absence of galanin (Fig. 5). Fold changes of cytokine/chemokine mRNA expression levels after exposure to galanin during differentiation and polarization compared to control samples without galanin treatment are summarized in an expression heat map (Fig. 6).

Macrophages differentiated for 6 days with GM-CSF in the presence of galanin (M0-GM + GAL-Mφ) and cultured for further 20 hours without any additional treatment (unpolarized) showed similar levels of expression of a range of cytokines and chemokines as unpolarized macrophages differentiated without galanin (M0-GM-Mφ) (Figs 5, 6, Supplementary Table S1). Only IL-18 mRNA levels differed significantly, showing a 1.2-fold reduction in the macrophages exposed to galanin during differentiation (p = 0.0106) (Fig. 6, Supplementary Table S1). Similarly, macrophages differentiated for 6 days with M-CSF in the presence of galanin (M0-M + GAL-Mφ) and cultured for 20 hours without further treatment showed a similar cytokine/chemokine expression profile as macrophages differentiated without galanin (M0-M-Mφ) (Fig. 6, Supplementary Table S1).

However, both M0-GM-Mφ and M0-GM + GAL-Mφ treated with 10 nM galanin for 20 hours after removal of the differentiation factors exhibited a ~2-fold decrease of IL-12p35 (p = 0.0082), TNFα (p = 0.0050), IL-10 (p = 0.0098), TGF-β (p = 0.0018), CXCL8 (p = 0.0310) and CCL3 (p = 0.0029) mRNA levels compared to untreated control cells (Figs 5, 6, Supplementary Table S1). The cytokine/chemokine profiles of M0-M-Mφ and M0-M + GAL-Mφ treated with 10 nM galanin for 20 hours showed no difference compared to control cells not treated with galanin post-differentiation (Fig. 6, Supplementary Table S1).

Interestingly, M1-GM + GAL-Mφ differentiated in the presence of 10 nM galanin expressed IL-1Ra (p = 0.0092) and CXCL8 (p = 0.0324) at a ~1.5-fold higher level compared to M1-GM-Mφ differentiated without galanin (Figs 5, 6, Supplementary Table S1). Furthermore, polarization to M1-GM-Mφ and M1-GM + GAL-Mφ in the presence of 10 nM galanin resulted in a 1.6- to 5-fold increase in the expression of CXCL8 (p = 0.0029), CCL2 (p = 0.0384), CCL3 (p = 0.0333) and CCL5 (p = 0.0044), and in a 1.5- to 2-fold increase in the expression of CCL22 (p = 0.0080), IL-10 (p = 0.0093), IL-1Ra (p = 0.0047), TGF-β (p = 0.0024) and TNFα (p = 0.0389) compared to polarization of M1-GM-Mφ and M1-GM + GAL-Mφ without galanin (Figs 5, 6, Supplementary Table S1).

Polarization to M2a-M-Mφ and M2a-M + GAL-Mφ in the presence of galanin significantly increased the levels of IL-18 (p = 0.0049), TNFα (p = 0.0342), TGF-β1 (p = 0.0332) and CCL2 (p = 0.0021) compared to M2a-M-Mφ and M2a-M + GAL-Mφ polarized without galanin (Fig. 6, Supplementary Table S1). Interestingly, only in M2a-M + GAL-Mφ expression of IL-10 (p = 0.001), IL-23p19 (p = 0.023) and CCL3 (p = 0.002) was increased upon polarization in the presence of galanin compared to polarization without galanin (Fig. 6, Supplementary Table S1).

The cytokine/chemokine profile of M2c-M + GAL-Mφ showed an increase of ~1.5-fold in CCL22 expression (p = 0.0212) compared to M2c-M-Mφ (Fig. 6, Supplementary Table S1). Furthermore, polarization to M2c-M-Mφ and M2c-M + GAL-Mφ in the presence of 10 nM galanin led to increased (~1.5-fold) mRNA levels of IL-1β (p = 0.0331), IL-1Ra (p = 0.0336), TGF-β1 (p = 0.0152), CXCL8 (p = 0.0299), CCL3 (p = 0.0107), CCL5 (p = 0.0185) and CCL22 (p = 0.0298) compared to polarization without galanin (Fig. 6, Supplementary Table S1). CCL2 expression was elevated ~2-fold (p = 0.001) in M2c-M + GAL-Mφ upon polarization in the presence of 10 nM galanin compared to polarization without galanin (Fig. 6, Supplementary Table S1).

### Galanin modulates chemokine secretion by macrophages

**Figure 7:**
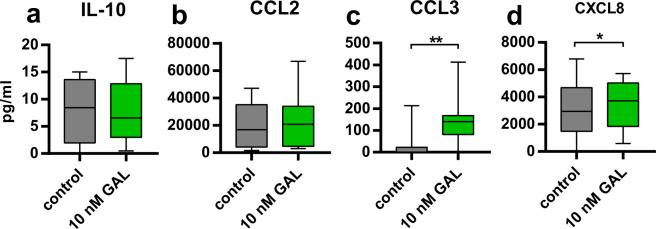
Cytokine/chemokine levels in the supernatant of macrophages (M0-GM + GAL-Mφ) cultured with or without galanin for 20 hours. The values are represented in box and whiskers plot format – min to max; n = 10. *p < 0.05, **p < 0.01.

As IL-10, TGF-β, CCL2, CCL3 and CXCL8 mRNA expression in GM-CSF-differentiated macrophages was highly affected by galanin treatment, we analyzed if galanin is also able to stimulate the secretion of these cytokines and chemokines into cell culture supernatants. TGF-β levels were under the detection limit (data not shown). We did not observe a difference in secreted IL-10 and CCL2 levels in M0-GM + GAL-Mφ upon galanin treatment for 20 hours (Fig. 7a,b). Interestingly, galanin stimulation resulted in increased CCL3 and CXCL8 levels in the supernatant of M0-GM + GAL-Mφ (Fig. 7c,d), although mRNA levels were reduced after 20 hours.

## Discussion

Receptors of diverse regulatory peptides such as CRH, Calcitonin gene-related peptide (CGRP), SP, α-Melanocyte-stimulating hormone (α-MSH), VIP, NPY and others were reported to be expressed by various immune cells^46^. Furthermore, nerve endings and different immune cells in primary and secondary lymph nodes release regulatory peptides^46,47^. These findings imply an essential role of regulatory peptides in controlling immune cell functions. Thus, diverse effects of regulatory peptides on immune cell migration, proliferation, maturation and cytokine/chemokine regulation have been described for PMNs, dendritic cells, monocytes, macrophages, NK cells and T cells^46–48^. Expression of the galanin peptide system parallels that of other regulatory peptides, in that leukocytes can serve as both a source and acceptor of galanin receptor ligands. Because macrophages secrete full-length galanin and express galanin receptors, an autocrine signaling mechanism can be assumed. Accordingly, immunocytes positive for EP1 (a marker for monocytes/macrophages) were shown to produce increased amounts of galanin during inflammation in rats^24^. In addition, galanin might also act as a paracrine factor, as various immune cells, including important macrophage-interacting cells like CD4^+^ T cells, express GAL_2_-R. GAL_2_-R signaling leads to activation of PKC, and several isoforms of PKC were reported to modulate T cell activation, polarization and survival, and also affect B cell and NK cell functions^18^. GAL_2_-R is expressed by diverse immune cells; nevertheless, subtype expression analysis of monocytes, B cells and T cells needs to be performed in future studies. As galanin affected the expression profile of macrophages in a subtype-specific manner and activation of PKC modulates T cell activation and polarization, we assume that a specific activation and/or T cell polarization could be facilitated or inhibited by galanin via GAL_2_-R. Similarly, a galanin-GAL_2_-R interaction on B cells could lead to a specific cell activation. The expression of specific galanin receptors and the combination of them on different types of immune cells might enable galanin to exert its effects in an immune cell type- and activation/tissue type-specific manner. However, expression of galanin, GAL_1_-R and GAL_3_-R (but not GAL_2_-R) and involvement of the galanin peptide in proliferation and apoptosis of immature rat thymocytes have already been reported^49^. An observed difference in receptor expression between immature rat thymocytes and human T cells isolated from peripheral blood^49^ may be due to species-specific differences or be related to the different developmental stages of the immune cells.

Macrophages expressed GAL_1_-R and GAL_2_-R, albeit only a part of M0-GM-Mφ showed membrane-associated GAL_1_-R and even less cells GAL_2_-R protein expression. The only partially membrane-associated GAL_1_-R and GAL_2_-R positive cells can be explained by a receptor internalization. It has been demonstrated that especially GAL_2_-R can be internalized upon ligand binding^4,50,51^. As macrophages already secrete high amounts of galanin into the supernatant galanin-receptor interaction could be followed by receptor internalization. In addition, GAL_1_-R and GAL_2_-R positive macrophages could have different characteristics as receptor negative cells, which has to be clarified in future studies. M0-M-Mφ did not reveal a clear GAL_1_-R positive protein expression; however, the cells exhibited an intracellular GAL_2_-R staining, which was also observed in GAL_2_-R overexpressing neuroblastoma cells^44^ and PC12 cells^51^. The lack of membrane-associated galanin receptors could explain the moderate response of M0-M-Mφ upon exogenous administered galanin. *In vivo* relevance of galanin receptor expression on macrophages was demonstrated by immunohistochemical staining of GAL_1_-R and GAL_2_-R on macrophages in human xanthelasma of the skin. These cholesterol deposits consist of foam cell-forming macrophages and it was reported that lipid oxidation inside the foam cells induced M-CSF synthesis^45^.

We cannot exclude that only a small population of macrophages were stimulated by galanin. However, the stimulation of this subgroup might have resulted in the secretion of cytokines and chemokines which then further stimulated neighboring cells.

A co-expression of both galanin receptors could result in a more flexible signaling cascade, as these receptors were reported to assemble into both homo- and heterodimers^52^. Interestingly, macrophage subtypes differed in the proportion of GAL_1_-R and GAL_2_-R expression and thus could have distinct signaling upon galanin exposure. GAL_1_-R and GAL_3_-R activation leads to inhibition of the second messenger cAMP and inactivation of PKA^4^, which can influence macrophage polarization^19–22^.

The importance of galanin ligand–receptor-dependent communication within inflammatory processes was previously demonstrated in diverse inflammatory animal models, including IBD, arthritis, dermatitis, psoriasis and pancreatitis^28–34^. Interestingly, the findings reveal a complexity in how the galanin system affects immune responses, in that galanin and its receptors were reported to exhibit both pro- and anti-inflammatory properties^28–34^. These biphasic properties might depend on the type and duration of inflammation and consequently the types of immune cells involved. The microenvironment or cytokine/chemokine milieu might also play a role. However, the impact of galanin on immune cell functions, especially on specific subsets, has received little attention up to now.

To our knowledge, the present study provides the first insight into how galanin affects diverse macrophage subtypes differently. Galanin was downregulated in macrophages by IFNγ (an important effector cytokine of T_h_1 responses) and LPS (M1-GM-Mφ), whereas IL-4 (M2a-M-Mφ), a main effector of T_h_2 responses, had no effect on galanin production by macrophages. Furthermore, these polarizations revealed different relative involvements of GAL_1_-R and GAL_2_-R in type-1 (M1-GM-Mφ) and type-2 (M2a-M-Mφ and M2c-M-Mφ) immune responses. This observation needs to be studied in more detail *in vivo* when considering galanin receptors as targets for therapeutic strategies in inflammatory diseases. The M2c subtype, in turn, showed similar levels of GAL_1_-R/GAL_2_-R and downregulation of galanin secretion, indicating fine-tuning of the galanin system during an immune challenge, especially as an immune response is not a static process. Interestingly, unpolarized type-2 macrophages (M0-M-Mφ) where unaffected by galanin stimulation though they expressed both GAL_1_-R and GAL_2_-R. Thus, an extra stimulus might be needed to change galanin functions, similar to what we observed in NK cells and PMNs^36,37^. Furthermore, secretion of chemokines CCL3 and CXCL8 was increased upon galanin treatment of unpolarized type-1 macrophages (M0-GM-Mφ), although gene expression after 20 hours was significantly downregulated. This indicates an initial boost of CCL3 and CXCL8 expression and downregulation at a later phase. Polarization of macrophages with IFNγ and LPS in the presence of galanin (M1-GM + GAL-Mφ) increased the gene expression of chemokines, including CCL3 and CXCL8, compared to the control polarized without galanin, which leads us to speculate that macrophages require a specific immune challenge to maintain their pro-inflammatory properties. The finding of increased CXCL8 production upon galanin stimulation was already reported in human keratinocytes^53^. CXCL8 mainly targets granulocytes like neutrophils or basophils via CXCR1 and CXCR2. CCL3 interact with CCR1 or CCR5 expressing cells including monocytes/macrophages, dendritic cells, granulocytes, NK cells but also subpopulations of T cells and B cells. Following, the augmented secretion of the chemokines CCL3 and CXCL8 by macrophages upon the activation of galanin receptor signaling they potentially influence leucocyte migration or perhaps other chemokine related functions^54^. Previously we observed biphasic properties of galanin affecting PMNs and NK cells^36,37^. Galanin was able to sensitize and desensitize murine PMNs toward CXCL1 depending on the galanin concentration^37^.

Thus, the pro- or anti-inflammatory properties of the galanin system depend on many factors, which have to be considered in future *in vitro* and *in vivo* studies. Alteration of chemokine expression and secretion by galanin indicates a role of galanin in immune cell migration, which was also reported for other regulatory peptides, such as the ability of SP to promote monocyte migration upon induction of chemokine expression^55^. Furthermore, similar to galanin, diverse regulatory peptides like SP, NPY or VIP can alter cytokine expression and secretion by immune cells^46^. SP was shown to upregulate TNFα secretion by mast cells^56^ and IL-1 and IL-6 secretion by monocytes^57^, and VIP was reported to exert an anti-inflammatory effect, inhibiting IL-12 secretion and IL-10 induction by LPS-induced macrophages^46^. NPY was shown to induce cell migration and secretion of IL-6 and IL-10 by monocyte-derived immature dendritic cells. Moreover, NPY promoted T_h_2 cell polarization^58^. Interestingly, CGRP was shown to modulate macrophage polarization by counter-regulating the expression of IL-1β and expression/activity of NACHT, LRR and PYD domain-containing protein 3 (NALP3), a marker for classic LPS-activated type-1 macrophages. Furthermore, CGRP and IL-4 co-stimulated the expression of IL-10, resistin-like beta (Fizz1) and mannose receptor C-type 1 (CD206) as well as the expression/activation of arginase 1, a marker for an alternative mode of type-2 macrophage activation, compared to IL-4 stimulation alone^59^. Similarly, SP was reported to support type-2 macrophage polarization^60^.

We conclude that the galanin system plays an important role in diverse immunoregulatory mechanisms, spanning from the early immune response to the later phase of inflammation, as we showed that galanin and its receptors are expressed and regulated in cells of both the innate and adaptive immune systems. Furthermore, galanin might affect immune cell migration, as chemokine expression and secretion by macrophages were clearly altered upon galanin treatment. In addition, galanin could modulate cell polarization, because it effected a more potent change in the cytokine/chemokine profile of type-1 macrophages than type-2 macrophages. Our study establishes that galanin is a potent modulator of cytokine and chemokine expression and thus a player in fine-tuning the macrophage-driven immune response.

## Materials and Methods

### Ethical statement

Experiments were conducted in accordance with the Helsinki Declaration of 1975 (revised 1983) and the guidelines of the Salzburg State Ethics Research Committee (AZ 209-11-E1/823-2006), being no clinical drug trial or epidemiological investigation. All patients signed an informed consent document for the use of surplus material for research purposes (regarding buffy coats) or concerning the surgical intervention (regarding human tissue samples). Furthermore, the study did not extend to examination of individual case records. Patient anonymity was ensured at all times.

### Immune cell isolation and differentiation

All immune cells were isolated from surplus buffy coat material from healthy donors. Buffy coats were generated and preselected as recently described^36^. Peripheral blood mononuclear cells (PBMCs) were isolated by Ficoll-Paque 1.077 g/l (GE Healthcare, Chicago, IL, USA) gradient centrifugation. Human PMNs and NK cells were isolated as described recently^36,37^. CD3^+^ T cells were positively selected from PBMCs using an anti-CD3-FITC and anti-FITC MultiSort Kit (Miltenyi Biotec, Bergisch Gladbach, Germany) following the manufacturer’s instructions. After removal of MultiSort MicroBeads, CD4^+^ and CD8^+^ T cells were separated using CD4 MicroBeads (Miltenyi Biotec). B cells were positively selected from PBMCs using CD19 MicroBreads (Miltenyi Biotec). CD14^+^ monocytes were isolated by using the Human Pan Monocytes Isolation Kit (Miltenyi Biotec).

Monocytes (5 × 10^5^ cells/well) were seeded in RPMI-1640 medium containing 10% fetal bovine serum (FBS) (Thermo Fisher Scientific, Waltham, MA, USA), 2 mM GlutaMAX (Thermo Fisher Scientific), 10 mM HEPES (Sigma-Aldrich, St. Louis, MO, USA) and 1x penicillin-streptomycin-amphotericin b mixture (Lonza, Basel, Switzerland) in a 24-well plate. Monocytes were differentiated for 6 days with 25 ng/ml GM-CSF (Miltenyi Biotec) to generate M0-GM-Mφ and 25 ng/ml M-CSF (Miltenyi Biotec) to generate M0-M-Mφ with or without 10 nM synthetic galanin (GL Biochem, Shanghai, China)^39,61–65^. The medium, including the supplements, was renewed every second day. After 6 days of differentiation, the medium was exchanged and M0-GM-Mφ and M0-M-Mφ were treated with or without 10 nM galanin for 20 hours. Furthermore, M0-GM-Mφ were polarized to M1 macrophages with 20 ng/ml IFNγ (Miltenyi Biotec) and 100 ng/ml LPS from *Escherichia coli* O26:B6 (Sigma-Aldrich) (M1-GM-Mφ) with or without 10 nM galanin. M0-M-Mφ were polarized to M2a macrophages with 20 ng/ml IL-4 (Miltenyi Biotec) (M2a-M-Mφ) with or without 10 nM galanin or to M2c macrophages with 20 ng/ml IL-10 (Miltenyi Biotec) (M2c-M-Mφ) with or without galanin^39,61–65^. Macrophages differentiated with GM-CSF or M-CSF in the presence of galanin were named M0-GM + GAL-Mφ, M1-GM + GAL-Mφ, M0-M + GAL-Mφ, M2a-M + GAL-Mφ and M2c-M + GAL-Mφ accordingly.

### Determination of mRNA levels of the galanin system and cytokines/chemokines by quantitative PCR

RNA of primary macrophages was isolated with Tri Reagent (Molecular Research Center, Inc., Cincinnati, OH, USA) according to the manufacturer’s instructions. Two micrograms of human RNA were used to generate cDNA by using maxima reverse transcriptase (Thermo Fisher Scientific) following the manufacturer’s protocol. Expression levels were quantified via qPCR using SYBR green SuperMix (BioRad, Hercules, CA, USA). The amplification was performed for 40 cycles (97 °C for 15 sec, 63 °C for 30 sec and 72 °C for 10 sec) with specific primers for the genes of interest (Supplementary Table S2). Relative expression levels of all genes were calculated by the ΔΔ quantification cycle (Cq) to the housekeeping gene RPL27 [2^−(Cq of the gene of interest−Cq of the housekeeping gene RPL27)^].

### Determination of galanin receptor protein expression in primary macrophages by immunofluorescence microscopy

Monocytes (2.5 × 10^5^ cells/well) were seeded in 8-well chamber slides (Corning Inc., Corning, NY, USA) and differentiated to M0-GM + GAL-Mφ and M0-M + GAL-Mφ as described before. Afterwards, the cell medium was exchanged with HBSS +/+ (Thermo Fisher Scientific) containing 20 mM HEPES and macrophages starved for two hours. The cells were washed with phosphate buffered saline (PBS) and fixed for 20 min by PBS containing 4% paraformaldehyde (PFA) at room temperature (RT). Fixed cells were washed twice with PBS for 5 minutes at RT and further incubated in 0.05 M tris-buffered saline (TBS) containing 5% donkey serum (Sigma–Aldrich), 1% bovine serum albumin (BSA; Sigma–Aldrich) and 0.5% Triton X-100 (Merck, Darmstadt, Germany) for 1 hour at RT. After blocking, cells were washed 3 times with 0.05 M TBS for 5 min at RT and incubated overnight with antibodies against human GAL_1_-R (GTX108207, 1:100; Genetex, Irvine, CA, USA), human GAL_2_-R (customized: S4510-1, 1:100; Proteintech, Manchester, UK) and rabbit-IgG (isotype control, I5006, 1:300; Sigma-Aldrich) diluted in 0.05 M TBS containing 1% BSA and 0.5% Triton X-100 at RT (Primary antibody concentrations: 3 µg/ml). The next day, cells were washed 3 times and incubated for one hour with an anti-rabbit, Alexa Fluor 555 conjugated antibody (1:1000; Thermo Fisher Scientific) at RT. Afterwards, cells were washed 3 times and cell nuclei were counterstained with 4′,6-Diamidino-2 phenylindol dihydroclorid (DAPI, 1:4000, Merck) for 10 min at RT. Samples were washed 2 times and sections were embedded in TBS-glycerol (1:1 at pH 8.6). For immunofluorescence microscopy a confocal laserscanning unit (Axio ObserverZ1 attached to LSM710, Zeiss, Oberkochen, Germany; 40x oil immersion objective lens, with numeric apertures 1.4, Zeiss) was used. Stained macrophages were imaged using the appropriate filter settings for Alexa Fluor 555 (543 nm excitation, coded red) and DAPI (345 nm excitation, coded blue). All images presented here represent confocal images in single optical section mode.

### Determination of *in vivo* galanin receptor protein expression by immunohistochemistry

Formalin-fixed paraffin-embedded (FFPE) tissue blocks of a skin biopsies of a patient with a xantelasma were analyzed for GAL_1_-R and GAL_2_-R by immunohistochemistry. All reagents used in immunohistochemistry were obtained from Leica (Wetzlar, Germany). FFPE tissues were sectioned to 5 μm, mounted and dried at 60 °C for 1 h. After deparaffinization and rehydration, heat-induced epitope retrieval was performed with BOND Epitope Retrieval solution for 20 min at 100 °C. Primary antibodies against human GAL_1_-R (GTX108207, 1:200) and GAL_2_-R (customized: S4510-1, 1:200 were diluted in BOND Primary Antibody Diluent. Slides were incubated with the primary antibody for 15 min at RT. After washing with Wash Solution, the Novolink Max polymer (second antibody) was applied for 30 min at RT. Another washing step was followed by visualization with Mixed Red Refine for 10 min at RT followed by another 5 min at RT. Slides were washed in deionized water and counterstained in Mayer’s hemalum solution (VWR, Radnor, PA, USA) for 10 min at RT. Slides were washed in deionized water, wash solution and 96% ethanol and after dehydration with 2-propanol, the slides were incubated in xylene and mounted. Digital micrographs were taken with an Olympus BX43 (Olympus Corporation, Tokyo, Japan).

### Determination of galanin peptide secretion by macrophage subtypes using an in-house ELISA kit

Complete mini protease inhibitor cocktail (Roche, Basel Switzerland) was added to cell culture supernatants before storage at −80 °C. Cell supernatants were analyzed for IL-10, TGF-β, CCL2, CCL3 and CXCL8 concentrations using specific ELISA kits (Thermo Fischer Scientific). For detection of secreted full-length galanin in supernatants of macrophages, an in-house galanin sandwich ELISA was used as described recently^43^.

### Statistical analysis

Statistical analyses were performed by using GraphPad Prism 7 (GraphPad Software, Inc., San Diego, CA, USA). Significances of galanin secretion and expression were calculated for GM-CSF-differentiated macrophages with a paired t-test and M-CSF-differentiated macrophages with a matched one-way ANOVA followed by Tukey’s multiple comparison test. Correlation between galanin secretion and mRNA levels was computed using Pearson correlation coefficients. Statistical analysis of galanin receptor expression in macrophage subtypes was performed by using a two-way ANOVA (Experimental design: repeated measures by both factors; receptor and cell subtype) followed by a Tukey multi comparison test. Cytokine and chemokine expression data sets were analyzed by using a two-way ANOVA (Experimental design: repeated measures by both factors; differentiation and polarization) followed by a Sidak multi comparison test when the interaction of main factors was significant. Data sets generated from ELISA were tested for Gaussian distribution using the D’Agostino-Pearson omnibus normality test and further analyzed for significance with a paired t test (if the values passed the normality test) or a Wilcoxon matched-pairs signed rank test (if the values did not pass the normality test).

## Supplementary information

SI-GAL-Macrophages

## Data Availability

The datasets generated during and/or analyzed during the current study are available from the corresponding author on reasonable request.
