# Supplementary material for: Galanin is a potent modulator of cytokine and chemokine expression in human macrophages
Source: Sci Rep. 2019 May 10;9:7237. doi: 10.1038/s41598-019-43704-7 (PMC6510899; doi:10.1038/s41598-019-43704-7)
Supplement: Supplementary file 1 — SI-GAL-Macrophages [file 41598_2019_43704_MOESM1_ESM.pdf]

Supplementary Information

Galanin is a potent modulator of cytokine and chemokine expression in human macrophages

Andreas Koller, Susanne Maria Brunner, Rodolfo Bianchini, Andrea Ramspacher, Michael Emberger, Felix Sternberg, Sandra Schlager and Barbara Kofler

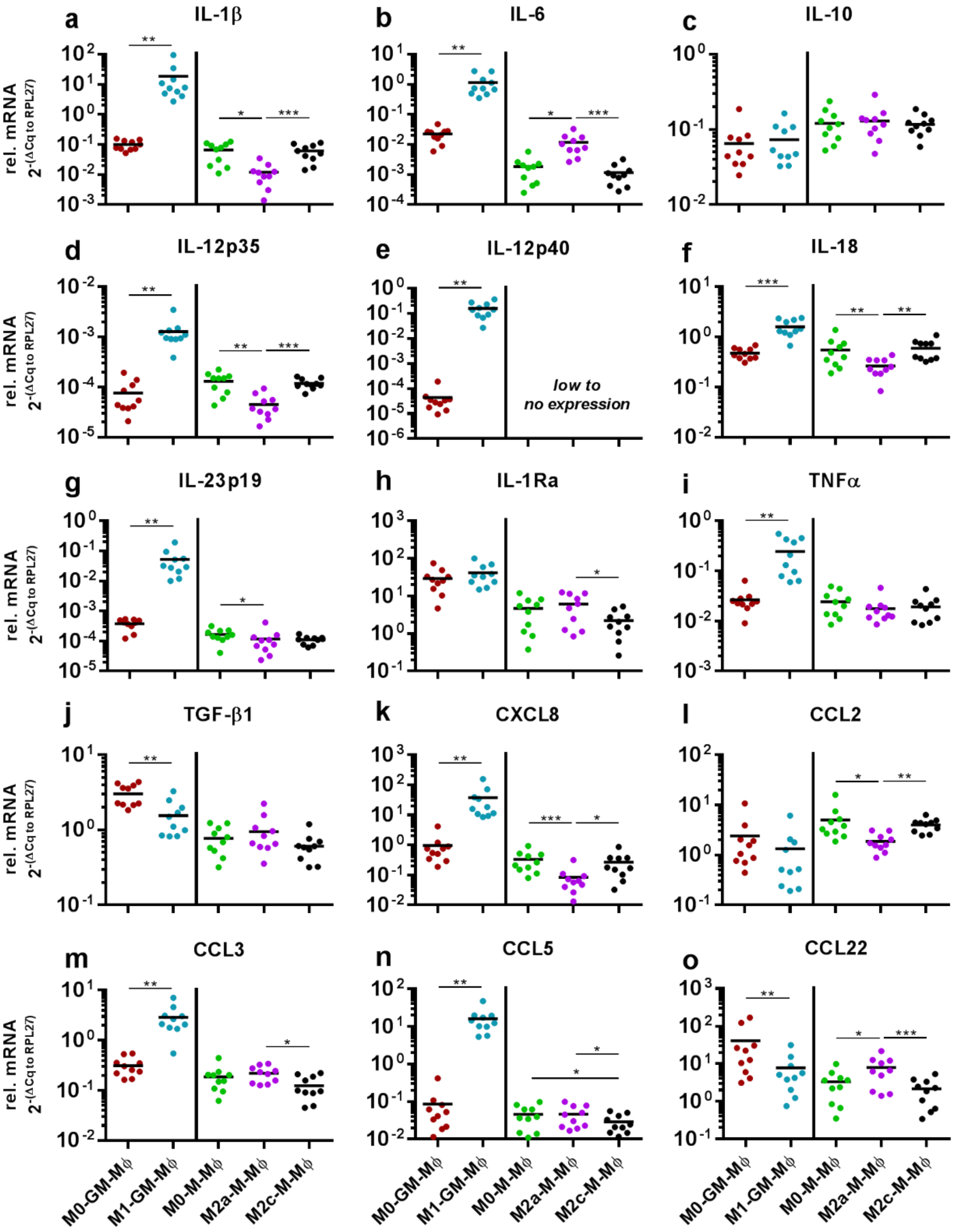

Supplementary Figure S1. Relative (**ΔΔ**Cq to housekeeping gene RPL27) mRNA levels of macrophage subtypes. Macrophages differentiated with GM-CSF and polarized without (M0-GM-Mφ) or with IFNγ+LPS (M1-GM-Mφ) and macrophages differentiated with M-CSF and polarized without (M0-M-Mφ) and with IL-4 (M2a-M-Mφ) or IL-10 (M2c-M-Mφ) were analyzed for expression levels of IL-1β (a), IL-6 (b), IL-10 (c), IL-12p35 (d), IL-12-p40 (e), IL-18 (f), IL-23p19 (g), IL-1Ra (h), TNFα (i), TGF-β1 (j), CXCL8 (k), CCL2 (l), CCL3 (m), CCL5 (n) and CCL22 (o)

by qPCR. Data sets of GM-CSF-differentiated macrophages (M0-GM-Mφ and M1-GM-Mφ) were tested for Gaussian distribution using the D'Agostino - Pearson normality test and further analyzed for significance with a paired t-test (if the values passed the normality test) or a Wilcoxon matched-pairs signed rank test (if the values did not pass the normality test). Data sets of M-CSF-differentiated macrophages (M0-M-Mφ, M2a-M-Mφ and M2c-M-Mφ) were tested for Gaussian distribution using the D'Agostino - Pearson normality test and further analyzed with a matched one-way ANOVA followed by Tukey's multiple comparison test (if the values passed the normality test) or a Friedman Repeated Measures Analysis of Variance on Ranks followed by Dunn's multiple comparison test (if the values did not pass the normality test). $^{*}p < 0.05$, $^{**}p < 0.01$, $^{***}p < 0.001$

Supplementary Table S1: Mean change in % ± SD of cytokine/chemokine expression in macrophage subtypes upon galanin treatment during differentiation and/or polarization to respective control samples differentiated and polarized without galanin (Supplementary Fig. S1).

Cell type	**M0**	**M0**	**M0**	**M1**	**M1**	**M1**	**M0**	**M0**	**M0**	**M2a**	**M2a**	**M2a**	**M2c**	**M2c**	**M2c**
Differentiation (6 days)	**GM+GAL Mφ**	**GM Mφ**	**GM+GAL Mφ**	**GM+GAL Mφ**	**GM Mφ**	**GM+GAL Mφ**	**M+GAL Mφ**	**M Mφ**	**M+GAL Mφ**	**M+GAL Mφ**	**M Mφ**	**M+GAL Mφ**	**M+GAL Mφ**	**M Mφ**	**M+GAL Mφ**
Polarization (20 hours)				+ 20 ng/ml IFNγ + 100 ng/ml LPS						+ 20 ng/ml IL-4			+ 20 ng/ml IL-10		
		+ 20 hours 10 nM GAL			+ 20 hours 10 nM GAL			+ 20 hours 10 nM GAL			+ 20 hours 10 nM GAL			+ 20 hours 10 nM GAL	
**IL-1β**	94.7±42.8	73.6±52.2	72.9±35.0	138.6±70.2	137.0±52.5	199.6±108.0	149.5±122.2	116.4±37.0	165.5±100.4	139.2±71.9	89.0±15.8	181.9±114.6	145.1±96.5	94.8±31.3	203.5±152.4
	Diff: 0.3848; Treat: 0.0863; D×T:0.9331			*Diff: 0.9507; Pol: 0.0194; D×P: 0.2987*			*Diff: 0.2307; Treat: 0.2927; D×T: 0.3785*			*Diff: 0.4004; Pol: 0.6395; D×P: 0.0757*			*Diff: 0.0761; Pol: 0.0331; D×P: 0.1491*		
**IL-6**	81.0±46.4	71.8±52.2	55.6±36.7	124.1±66.0	118.9±49.5	160.2±87.6	201.6±251.4	118.2±44.3	275.4±227.3	103.1±39.5	85.5±24.6	136.3±69.2	138.9±87.7	102.8±59.1	203.7±140.9
	Diff: 0.6572; Treat: 0.1077; D×T:0.6635			*Diff: 0.1693; Pol: 0.1497; D×P: 0.8654*			*Diff: 0.0674; Treat: 0.2715; D×T: 0.2977*			*Diff: 0.3957; Pol: 0.7173; D×P: 0.0354*			*Diff: 0.0632; Pol: 0.1232; D×P: 0.0838*		
**IL-10**	93.9±76.5	52.4±31.0	41.7±27.5	126.7±71.8	148.5±53.8	200.7±103.4	109.7±60.2	107.8±41.5	110.7±53.0	90.1±36.1	147.0±61.6	162.1±64.8***	104.2±71.5	115.7±35.5	133.6±47.1
	Diff: 0.1674; Treat: 0.0098; D×T:0.4488			*Diff: 0.2540; Pol: 0.0093; D×P: 0.8075*			*Diff: 0.9249; Treat: 0.7751; D×T: 0.5407*			*Diff: 0.6633; Pol: 0.0001; D×P: 0.0227*			*Diff: 0.6632; Pol: 0.0513; D×P: 0.3086*		
**IL-12p35**	65.7±51.3	51.3±28.2	44.1±29.3	154.9±136.1	128.8±50.5	165.0±101.5	113.6±67.9	127.7±132.2	129.4±60.4	82.4±53.3	136.5±96.5	144.3±91.1	103.0±68.9	100.5±42.6	136.0±52.4
	Diff: 0.0556; Treat: 0.0082; D×T: 0.0695			*Diff: 0.2127; Pol: 0.0723; D×P: 0.8715*			*Diff: 0.8677; Treat: 0.5117; D×T: 0.3029*			*Diff: 0.9935; Pol: 0.1197; D×P: 0.1658*			*Diff: 0.2393; Pol: 0.2509; D×P: 0.4550*		
**IL-12p40**	148.3±148.2	176.6±280.3	95.8±107.9	138.0±77.5	108.9±50.2	188.2±99.2	***low to no expression***			***low to no expression***			***low to no expression***		
	Diff: 0.5946; Treat: 0.2561; D×T: 0.8617			*Diff: 0.0812; Pol: 0.0774; D×P: 0.2119*											
**IL-18**	77.4±22.2	66.9±40.4	53.8±24.9	105.9±67.6	127.6±54.2	148.3±86.2	95.3±42.9	110.5±39.5	109.8±51.8	97.1±33.9	136.1±73.8	140.6±51.3	109.3±62.7	95.9±28.2	132.3±59.7
	Diff: 0.0106; Treat: 0.0018; D×T: 0.2351			*Diff: 0.7704; Pol: 0.0396; D×P: 0.5384*			*Diff: 0.2846; Treat: 0.8332; D×T: 0.1766*			*Diff: 0.8327; Pol: 0.0049; D×P: 0.6270*			*Diff: 0.5886; Pol: 0.1795; D×P: 0.1006*		
**IL-23p19**	84.4±55.8	71.2±71.4	72.0±76.8	163.2±144.4	109.0±34.2	156.9±99.3	131.0±123.5	123.5±55.3	151.3±152.9	91.4±50.6	107.5±78.4	195.7±157.1*	97.02±59.2	108.8±54.7	159.2±77.1
	Diff: 0.2188; Treat: 0.0858; D×T: 0.2039			*Diff: 0.6438; Pol: 0.4439; D×P: 0.8022*			*Diff: 0.3515; Treat: 0.4823; D×T: 0.5340*			*Diff: 0.5556; Pol: 0.4958; D×P: 0.0139*			*Diff: 0.0748; Pol: 0.2157; D×P: 0.0989*		
**IL-1Ra**	81.3±40.8	54.7±43.5	50.2±40.7	148.5±84.4	181.1±99.5	276.9±179.2	114.5±66.3	109.7±69.4	127.5±104.4	184.3±121.0	107.4±37.6	220.2±186.0	156.8±84.7	123.9±67.5	211.3±112.9
	Diff: 0.2436; Treat: 0.0064; D×T: 0.1682			*Diff: 0.0092; Pol: 0.0047; D×P: 0.5451*			*Diff: 0.8550; Treat: 0.7163; D×T: 0.9035*			*Diff: 0.2389; Pol: 0.5838; D×P: 0.6086*			*Diff: 0.1569; Pol: 0.0336; D×P: 0.2509*		
**TNFα**	100.8±70.2	64.8±40.1	48.8±27.3	139.9±95.3	136.3±67.2	240.9±262.4	129.2±83.4	105.5±32.9	158.7±76.8	122.6±50.5	116.9±47.3	152.9±57.9	123.4±84.2	99.7±39.7	153.8±74.3
	Diff: 0.4416; Treat: 0.0050; D×T: 0.8698			*Diff: 0.1785; Pol: 0.0389; D×P: 0.3687*			*Diff: 0.1227; Treat: 0.2948; D×T: 0.1011*			*Diff: 0.1110; Pol: 0.0342; D×P: 0.9675*			*Diff: 0.2676; Pol: 0.0900; D×P: 0.0909*		
**TGF-β1**	85.4±50.6	60.9±47.1	45.3±20.1	104.3±40.8	162.3±59.3	234.6±216.3	108.5±53.5	95.7±28.8	95.4±35.1	99.2±35.5	124.8±52.0	132.1±54.3	112.2±66.1	115.0±33.1	160.8±73.1
	Diff: 0.0824; Treat: 0.0018; D×T: 0.5642			*Diff: 0.7902; Pol: 0.0024; D×P: 0.5577*			*Diff: 0.9375; Treat: 0.0673; D×T: 0.9755*			*Diff: 0.6707; Pol: 0.0332; D×P: 0.5145*			*Diff: 0.2395; Pol: 0.0152; D×P: 0.1254*		
**CXCL8**	82.7±39.0	45.2±39.5	41.9±25.4	152.0±63.6	333.6±192.9	499.1±329.5	101.5±46.9	89.1±32.0	111.9±51.9	105.8±61.4	128.6±61.7	254.4±218.2	107.8±64.0	140.5±35.4	189.9±81.1
	Diff: 0.3427; Treat: 0.0310; D×T: 0.3134			*Diff: 0.0324; Pol: 00029; D×P: 0.9595*			*Diff: 0.2738; Treat: 0.8691; D×T: 0.3641*			*Diff: 0.1643; Pol: 0.0692; D×P: 0.1698*			*Diff: 0.0771; Pol: 0.0299; D×P: 0.2116*		
**CCL2**	66.1±32.6	54.5±43.9	36.9±21.0	133.2±81.0	180.4±69.0	308.4±225.0	131.7±78.8	90.8±31.6	114.6±55.5	102.3±37.7	158.2±65.9	170.5±58.9	112.1±58.9	115.2±34.7	222.7±78.2***
	Diff: 0.1311; Treat: 0.0646; D×T: 0.2154			*Diff: 0.1036; Pol: 0.0384; D×P: 0.8802*			*Diff: 0.6464; Treat: 0.2528; D×T: 0.4660*			*Diff: 0.3316; Pol: 0.0021; D×P: 0.3184*			*Diff: 0.0032; Pol: 0.0026; D×P: 0.0067*		
**CCL3**	79.4±28.1	48.6±35.0	47.2±27.2	284.7±490.5	164.2±81.6	434.8±522.2	118.5±50.5	94.8±39.3	111.5±44.9	92.8±24.6	106.9±32.2	140.4±59.7**	118.7±69.2	104.9±29.2	183.1±84.1
	Diff: 0.1620; Treat: 0.0029; D×T: 0.1522			*Diff: 0.0522; Pol: 0.0333; D×P: 0.2321*			*Diff: 0.4380; Treat: 0.2900; D×T: 0.5005*			*Diff: 0.4015; Pol: 0.0179; D×P: 0.0413*			*Diff: 0.1006; Pol: 0.0107; D×P: 0.0584*		
**CCL5**	101.7±61.6	64.2±45.6	55.3±37.0	113.7±42.3	235.1±120.4	350.1±222.0	120.6±69.4	109.9±58.1	118.2±73.1	102.3±44.9	108.3±49.5	169.3±92.0	139.4±90.8	125.5±58.5	195.6±88.2
	Diff: 0.2509; Treat: 0.0478; D×T: 0.7199			*Diff: 0.0685; Pol: 0.0044; D×P: 0.1089*			*Diff: 0.7650; Treat: 0.6896; D×T: 0.7689*			*Diff: 0.6131; Pol: 0.0561; D×P: 0.1875*			*Diff: 0.1056; Pol: 0.0185; D×P: 0.4038*		
**CCL22**	70.3±37.2	61.3±52.1	40.8±27.8	106.1±81.8	156.0±68.8	242.7±194.8	111.9±80.3	109.9±46.2	103.2±51.3	171.2±71.0	116.8±42.0	227.3±186.5	132.4±82.8	128.3±39.8	225.0±117.0
	Diff: 0.1048; Treat: 0.0769; D×T: 0.1979			*Diff: 0.6660; Pol: 0.0080; D×P: 0.0542*			*Diff: 0.7209; Treat: 0.5761; D×T: 0.8717*			*Diff: 0.1678; Pol: 0.2295; D×P: 0.6813*			*Diff: 0.0212; Pol: 0.0298; D×P: 0.0853*		

Statistics: Two-way ANOVA calculated with relative ΔΔCq values to RPL27; experimental design: repeated measures by both main factors: differentiation and polarization with galanin, Diff. = p values of differentiation with galanin versus differentiation without galanin, Treat. = p values of treatment with galanin versus treatment without galanin, Pol. = p values of polarization with galanin versus polarization without galanin; D×T = p values of interaction of differentiation and treatment with galanin versus without galanin, D×P = p values of interaction of differentiation and polarization with galanin versus without galanin. Significant main factors or interactions are colored in blue. Multiple comparison test if the interaction of the two main factors was significant: Sidak. *$p < 0.05$, **$p < 0.01$, ***$p < 0.001$ versus control polarized without galanin.

significant decrease

**Not significant**
**< 1.5 fold**
**> 1.5 fold < 2 fold**
**> 2 fold**

significant increase

**+ < 1.5 fold**
**+ > 1.5 fold < 2 fold**
**+ > 2 fold < 3 fold**
**+ > 3 fold**

Supplementary Table S2. Primer sequences for qPCR.

	**Forward 5' – 3'**	**Reverse 5' – 3'**
**RPL27**	GCTGGAATTGACCGCTACC	TCTCTGAAGACATCCTTATTGACG
**Galanin**	CTGCTCGCCTCCCTCCTC	TGTCGCTGAATGACCTGTG
**GALP**	GCTGGACCCTCAATAGTGC	CAAACGTCTCCATCACATTCC
**Spexin**	TGAAAGGGGCACAGGGTCGC	AGTTGGGGATTTGGGCTTCGTC
**GAL_1-R**	TCTGCTTCTGCTATGCCAAGG	AGAGATGGATGATGTGGTGCG
**GAL_2-R**	GCCGACCTGTGTTTCATCC	GGAGTGCAGCGGGTAG
**GAL_3-R**	TTACGCTGGCTGCTGTCTCC	CGGTGCCGTAGTAGCTGAGGTA
**IL-1β**	AGGCTGCTCTGGGATTCTCTTCA	CCATCCAGAGGGCAGAGGTCCA
**IL-6**	AGATGTAGCCGCCCCACACAG	CCAGTGCCTCTTTGCTGCTTTCA
**IL-10**	GACCCAGACATCAAGGCGCA	CATTCTTCACCTGCTCCACGGC
**IL-12p35**	GTTCCCATGCCTTCACCACTCC	GGCCTCCACTGTGCTGGTTTTA
**IL-12p40**	TGGACTCTCCGTCCTGCCCA	ACCACCATTTCTCCAGGGGCAT
**IL-18**	CCAGATCGCTTCCTCTCGCA	TCCAGGTTTTCATCATCTTCAGCTA
**IL-23p19**	AGTGGGACACATGGATCTAAGAGAA	CAAGCAGAACTGACTGTTGTCCC
**IL-1Ra**	TCTCCTCCTCTTCCTGTTCCATTCA	TTCCCAAGAACAGAGCATGAGGC
**TNFα**	CCTGCTGCACTTTGGAGTGA	CTTGTCACTCGGGGTTCGAG
**TGF-β**	GATGTCACCGGAGTTGTGCGG	CGGTAGTGAACCCGTTGATGTC
**CXCL8**	TGACTTCCAAGCTGGCCGTG	TTCTGTGTTGGCGCAGTGTGG
**CCL2**	ATTCCCCAAGGGCTCGCTCAG	CTGCTTGGGGTCAGCACAGAT
**CCL3**	CATGGCTCTCTGCAACCAGTTCT	CCGGCTTCGCTTGGTTAGGAAG
**CCL5**	CTACTGCCCTCTGCGCTCCT	TCGGGTGACAAAGACGACTGCT
**CCL22**	CCTCCTTGCTGTGGCGCTTC	CAGGGGCAGACGGTAACGGA
